# Self-assessed health among Thai elderly

**DOI:** 10.1186/1471-2318-10-30

**Published:** 2010-05-28

**Authors:** Fariha Haseen, Ramesh Adhikari, Kusol Soonthorndhada

**Affiliations:** 1 Health System and Infectious Diseases Division, ICDDR,B, GPO Box 128, Dhaka 1000, Bangladesh; 2 Institute for Population and Social Research, Mahidol University, Salaya, Phutthamonthon, Nakhon Pathom 73170, Thailand; 3 Geography and Population Department, Mahendra Ratna Campus, Tribhuvan University, Kathmandu, Nepal

## Abstract

**Background:**

The ageing of the population is rapidly progressing in Thailand. Self-assessed health status can provide a holistic view of the health of the elderly. This study aims to identify the determinants of self-assessed health among older Thai people.

**Methods:**

The data for this study were drawn from a national survey of older persons conducted in 2007. Stratified two-stage random sampling was used for data collection. The analysis was restricted to the population aged 60 and above. The study used univariate, bivariate, and multivariate analysis procedures to analyze the data. Bivariate analysis was used to identify the factors associated with self assessment of health status. After controlling for other variables, the variables were further examined using multivariate analysis (binary logistic regression) in order to identify the significant predictors of the likelihood of reporting poor health.

**Results:**

Overall, 30,427 elderly people were interviewed in this study. More than half of the sampled respondents (53%) were aged 60-69 years and about one out of seven (13%) were aged 80 years or above. About three in five respondents (56%) reported that their health was either fair or very bad/bad. Logistic regression analysis found that age, education, marital status, working status, income, functional status, number of chronic diseases, and number of psychosocial symptoms are significant predictors in determining health status. Respondents who faced more difficulty in daily life were more likely to rate their health as poor compared to those who faced less such difficulty. For instance, respondents who could not perform 3 or more activities of daily living (ADLs) were 3.3 times more likely to assess their health as poor compared to those who could perform all the ADLs. Similarly, respondents who had 1, 2, or 3 or more chronic diseases were 1.8 times, 2.4 times, and 3.7 times, respectively, more likely to report their health as poor compared to those who had no chronic disease at all. Moreover, respondents who had 1-2, 3-4, or 5 or more psychosocial symptoms in the previous months were 1.6 times, 2.2 times, and 2.7 times, respectively, more likely to report poor health compared to those who did not have any psychosocial symptoms during the same period.

**Conclusion:**

Self-assessed poor health is not uncommon among older people in Thailand. No single factor accounts for the self-assessed poor health. The study has found that chronic disease, functional status, and psychosocial symptoms are the strongest determinants of self-assessed poor health of elderly people living in Thailand. Therefore, health-related programs should focus on all the factors identified in this paper to improve the overall well-being of the ageing population of Thailand.

## Background

One of the most frequently used measures of self-assessed health (SAH) status is a single question asking patients to rate their overall health on a scale from excellent to very poor or very good to very bad. This simple global question provides a useful summary of how patients perceive their overall health status [1]. Recently Jylha (2009) has mentioned that SAH differs from other health indicators and that it originates from an active cognitive process that is not constrained by formal rules or definitions. SAH can be described as careful cognitive consideration of different knowledge and evaluations that might follow a logical sequence of steps or stages [2], but it could also be based on multiple psychological processes [3]. SAH is a valid indicator to predict changes in health and mortality [4-9]. It is also predictive of other important health-related outcomes, such as health service utilization and functional ability in old age [3]. However, concern has been raised about the subjective measurement of health because of differences in older adult health across societies that might not be explained by covariates alone [2,7].

To address Thailand's rapidly ageing society, a special insurance scheme was developed for the elderly in 1992 which had introduced health care card, and in 2001 the government started a new scheme for the entire country in order to cover those who previously had no coverage. The new scheme, called the 30-baht healthcare scheme, has the user pay 30 baht per visit, with additional costs covered by the government. In addition, accessibility to health centers has also been improved [10]. These programs aim to improve elderly health in general. The last three surveys on older persons, conducted in 1994, 2002, and 2007, found that self-assessed health has improved steadily for both men and women as well as for both younger and older elderly persons in Thailand. In each survey women are less likely to report their health as good or very good than are men, and older persons are considerably less likely than younger elderly persons to do so [11]. In spite of these improvements, inequalities remain. Many older people have difficulty with essential daily activities, and have problems with disability and dependency [12]. Increasing numbers of elderly people suffer from chronic illness such as heart disease, cancer, and dementia. The most common causes of death of Thai elderly are cancer, heart disease, cerebrovascular disease, pneumonia, kidney disease, and diabetes [13], and there is an increasing pattern of suicide among the general population [10,13].

Most of the studies on SAH of older people have been conducted in Western countries to predict mortality [14-23]. Only a limited number of studies have examined the subjective measurement of older people in non-Western countries, including Thailand. Two studies in Thailand have examined the association of socioeconomic status and SAH [24,25], both of which found socioeconomic status to be a strong predictor of SAH. Yiengprugsawan, Lim, Carmichael, Sidorenko and Sleigh [24] focused on the history of chronic illness, recent illness, and hospital illness. Zimmer and Amornsirisomboon [25] studied chronic illness and functional status. Tangcharoensathien, Cheawchanwattana, Limwattananon, Vasavid, Lerkiatbundit and Boonperm [26] assessed the health of the Thai population and found that those who lived in the northern region, outside a municipality, had incomes in the poorest quintile, were included in the Universal Coverage (30-baht) scheme, got sick, were hospitalized, or had chronic diseases, they reported lower physical and mental health scores [26]. Another study discussed the importance of the objective measurement of the Thai elderly population [27]. Yet we still do not know completely what factors could explain the self-assessment of older people's health in Thailand. Especially there is a gap of knowledge on the effect of chronic diseases, functional limitations and psychosocial symptoms in the self-assessed health among Thai elderly. The psychosocial component is an essential component of quality of life for elderly. The present study aims to examine the effect of chronic diseases, functional status and psychosocial symptoms on the self-assessment of health among older Thai people. We hypothesize that chronic diseases, functional status, and psychosocial symptoms could be important predictors in the self-assessments of health of older people in Thailand.

## Methods

### Study design and data collection

Interest and concern regarding ageing issues is relatively recent in Thailand, but Thai government agencies, particularly the National Statistical Office (NSO) has recognized the need for adequate information to develop appropriate policies and programs to ensure the well-being of the Thai elderly. The NSO has conducted three nationally representative household surveys of older persons, in 1994, 2002, and 2007 [28-30]. These surveys collected information on socioeconomic conditions and living arrangements, employment and income, health status and health behavior, and the basic needs and attitudes toward social welfare, including social participation of the elderly in Thai society. This paper makes use of the 2007 National Survey of Older Persons, which collected data by using stratified two-stage sampling. The primary sampling units were blocks for municipal areas and villages for non-municipal areas. The secondary sampling units were private households selected by random sampling from the list of all enumerated households in each block or village of the first sampling. Survey data were collected from 79,509 sampled households, and every member of a participating household aged 50 and older was interviewed, covering a total of 56,502 persons. Data used in this paper are from the population aged 60 and older which covered 30,427 individuals. Data were weighted to represent the structure of the Thai older population using weighting factors provided by the NSO. All statistical analyses were carried out with SPSS version 11.5. This study was approved by the ethics committee of the NSO of Thailand.

### Variables

#### Self-assessed health (SAH)

In this survey the question was asked for SAH, "How was your health in past 7-days?". The question had five response categories (very good, good, fair, bad and very bad) to collect information on the reported self-assessed health status of the elderly. We categorized the response categories into two groups: "Good," which included "very good" and "good," and "Poor," which included "fair," "bad," and "very bad."

*Demographic variables *included age (60-69 years, 70-79 years, and 80 years and above), sex (male and female), place of residence (urban and rural), and marital status (married, separated/divorced, widowed, single).

*Economic variables *were average total income per year (100,000 baht or more, 30,000-99,999 baht, 10,000-29,999 baht, and less than 10,000 baht) and working status in the previous 7 days (work and did not work).

*Social variables *included education (higher than secondary level, secondary level, primary/elementary level, no schooling) and living arrangements (living with children, living with spouse, living alone and living with others, including relatives and non-relatives).

#### Chronic diseases

Individuals were asked about the presence of hypertension, heart disease, diabetes, cancer, stroke, and paralysis. One composite indicator, "chronic diseases condition" was developed from all the chronic diseases, resulting in 4 categories: had no chronic disease, had 1 chronic disease, had 2 chronic diseases, and had 3 or more chronic diseases.

#### Functional status

To measure the *functional status *the question was asked, "Can you perform these activities on your own?" Four activities of daily lining (ADLs) (eating, getting dressed, bathing/going to the toilet, and sitting) and five instrumental activities of daily living (IADLs) (ability to carry things weighing 5 kgs, walk 200-300 meters, walk up 2-3 flights of stairs, take a bus/ship alone; and calculate and use money correctly) were included in the question. A composite indicator, "functional status," was made from both ADL and IADL questions, after which three categories were derived: ability to do all ADLs and IADLs, difficulty with 1 or 2 ADLs and IADLs, and difficulty in 3 or more ADLs and IADLs.

#### Psychosocial symptoms

For psychosocial symptoms the question was asked, "How often did you experience the following symptoms last month?" Respondents were asked about seven symptoms: stress, unhappiness, moodiness, hopelessness, uselessness, lack of appetite, and loneliness. One composite index, "psychosocial symptoms," was created, resulting in four categories, divided into no symptom, 1-2 symptoms, 3-4 symptoms, and more than 5 symptoms.

## Results

Our analysis is confined to those who are aged 60 years or more. The data were weighted. Univariate, bivariate, and multivariate analysis were performed. Initially, univariate or descriptive analysis was used to describe the percentage of the respondents' sociodemographic characteristics. Bivariate analysis was performed to identify associated factors to self assess the health status. A chi-square test was used to test the association between the variables. The variables were further examined in the multivariate analysis (binary logistic regression) in order to identify the significant predictors of the likelihood of reporting poor health after controlling for other variables. During the process of analysis, multi-collinearity among the variables was assessed. As none of the variables were highly correlated, all the variables were included in the logistic model.

More than half sampled respondents (53%) were aged 60-69 years while about one out of seven (13%) were aged 80 years or above. Respondents from rural areas (58%) outnumbered those from urban areas (42%). Nearly three out of five respondents (57%) were female. Similarly, a large majority of the respondents (72%) had a primary/elementary level education. A notable proportion of the respondents (35%) were widowed. A majority of the respondents had worked during the seven days preceding the survey. Almost half the respondents reported that their average total income per year was 30,000 baht or more. Around 8.4% respondents lived alone. About two in five respondents (38%) reported that they had difficulty in at least one activity in daily living. Moreover, more than two in five respondents (44%) reported that they had at least one chronic disease. More than two-thirds (69%) of the respondents reported that they had at least one symptom of psychosocial problems (Table 1). Furthermore, 40.6% reported that their health was good but only 3.3% said "very good." About three in five respondents (56%) reported that their health was either fair, bad, or very bad (table not shown).

**Table 1:** Characteristics of the elderly population according to their perception regarding their physical health

		% of respondents	Self assessment of health		N
				
			Good health	Poor health	
Age group***	60-69 years	53.0	52.3	47.7	16,131
	70-79 years	34.0	37.1	62.9	10,355
	80 years or +	13.0	27.0	73.0	3,941

Sex***	Male	43.0	49.2	50.8	13,088
	Female	57.0	39.8	60.2	17,339

Place of residence**	Rural	57.7	44.7	55.3	17,558
	Urban	42.3	42.7	57.3	12,869

Level of education ***	Higher than secondary level	4.1	68.1	31.9	1,246
	Secondary level	7.2	56.0	44.0	2,192
	Primary/elementary level	72.1	43.4	56.6	21,949
	No schooling	16.6	34.8	65.2	5,040

Marital status***	Married	59.3	48.3	51.7	18,050
	Separated/divorced	2.5	41.6	58.4	747
	Widowed	34.6	36.7	63.3	10,534
	Single	3.6	41.6	58.4	1,096

Working status during the past 7 days ***	Work	65.4	57.2	42.8	10,523
	Do not work	34.6	36.8	63.2	19,904

Average total income per year ***	100,000 Baht or more	16.0	55.8	44.2	4,867
	30,000-99,999 Baht	33.4	48.4	51.6	10,148
	10,000-29,999 Baht	34.2	40.3	59.7	10,391
	Less than 10,000 Baht	16.4	30.4	69.6	4,995

Living arrangement ***	Living with children	17.7	44.6	55.4	5,393
	Spouse	16.3	48.2	51.8	4,966
	Alone	8.4	42.4	57.6	2,568
	Other	57.5	42.6	57.4	17,500

Number of chronic diseases ***	No	56.1	53.3	46.7	17,073
	1 disease	29.2	35.5	64.5	8,885
	2 diseases	11.5	26.8	73.2	3,506
	3 or more diseases	3.2	16.0	84.0	963

Functional status ***	Able to do all ADLs	61.8	55.3	44.7	18,805
	Difficulty in 1 and 2 ADLs	18.1	34.5	65.5	5,522
	Difficulty in 3 or more ADLs	20.0	17.2	82.8	6,100

Psychosocial symptoms ***	No symptom	31.3	59.5	40.5	9,513
	1-2 symptoms	29.5	44.0	56.0	8,991
	3-4 symptoms	20.7	35.1	64.9	6,298
	5 or more symptoms	18.5	27.0	73.0	5,625

	**Total**	**100.0**	**43.9**	**56.1**	**30,427**

• Note: *** = p < .001, ** = p < .01, * = p < .05; 1$: Baht 33.00

The study found that self assessment of health is significantly associated with age, sex, place of residence, level of education, marital status, working status within the week, annual income, living arrangement, functional status, number of chronic diseases and psychosocial problems. A significantly higher proportion of respondents aged 80 years or above (73%) compared with only about half the respondents (48%) aged 60-69 reported that their health was poor. A higher proportion of urban respondents (57%) than rural (55%) reported that their health was poor. Similarly, a higher proportion of female (60%) than male (51%) considered their health was poor. Education has a negative effect on self assessment of health. For example, 65% of the participants who were illiterate classified their health as poor compared to 32% of those who had more than secondary education. A higher proportion of the single (58%), separated/divorced (58%), and widowed (63%) respondents perceived their health to be poor compared to married respondents 52%) (Table 1). A significantly lower proportion of rich respondents (44%) (with an annual income of 100,000 baht or more) reported that their health was poor than did the poorest respondents (70%) (with an annual income <10,000 baht). Moreover, the percentage of those reporting poor health was significantly lower (52%) among those who lived with a spouse than it was for others (55-58%). As expected, a significantly higher percentage of the respondents who had difficulty with three or more ADL (83%) reported their health as poor compared to those who were able to do all daily living activities. Similarly, a very high percentage (84%) of the respondents who had three or more chronic diseases mentioned that they had poor health compared to those who didn't have any chronic disease. Similarly, psychosocial problems had a positive effect on a self-assessment of poor health, with 73% of those who had five or more psychosocial symptoms reporting poor health compared to about two-fifths (41%) of those who had none of the psychosocial symptoms (Table 1).

Logistic regression analysis was used to measure the strength of the association between various demographic and socio-economic characteristics, functional status, chronic disease, and psychosocial problems and the probabilities of reporting poor health status. Four models were used in the analysis. The first model contained the sociodemographic variables. In the second and third models, the variables "chronic disease" and "functional status" respectively, were added. In the fourth model, the final model of the analysis, the variable "psychosocial symptoms" was added. Almost all variables which were significant in the model 1, model 2 and model 3 retained their significance level after inclusion of the variable of chronic diseases, functional status and psychosocial symptoms respectively. However, the reduction of the odds ratios in most of the variables indicated that the variables such as number of chronic diseases, functional status and psychosocial symptoms were also important predicators of self assessed health. The analysis found that respondents aged 70-79 years and 80 years or above were 22% (OR = 1.22) and 30% (OR = 1.30), respectively, more likely to report poor health compared to those who were aged 60-69 years. Similarly, those respondents who had only a primary/elementary level of education or no schooling at all were twice as likely to assess themselves as having poor health than were respondents with a secondary or higher education. Similarly, single respondents were more likely to assess their health as poor (OR = 1.24) compared to the respondents who were married. On the other hand, widowed respondent were less likely to assess their health as poor (OR = 0.92) compared to the respondents who were married. Moreover, respondents who did not work during the seven days preceding the survey were more likely to report (OR = 1.36) poor health than were those who worked during that time. Similarly, the poorest respondents were more likely to assess their health as poor than were the richest people. Furthermore, respondents who had one, two, or three or more chronic diseases were 1.8 times, 2.4 times, and 3.7 times, respectively, more likely to report their health as poor compared to those who did not have any chronic disease. Similarly, respondents who had more difficulty in daily living were more likely to rate their health as poor compared to those who had no such difficulty. For instance, respondents who had difficulty in performing three or more ADLs were 3.3 times more likely to assess themselves as possessors of poor health compared to those who no such difficulty. Moreover, respondents who had 1-2, 3-4, or 5 or more psychosocial symptoms in the previous month were, respectively, 1.6 times, 2.2 times, and 2.7 times more likely to report poor health compared to those who had no psychosocial symptoms in previous month (Table 2).

**Table 2:** Adjusted odds ratio (OR) of reported poor health status in Thai elderly people, by selected variables

		Odds Ratio			
		
		Model 1	Model 2	Model 3	Model 4
Age group	60-69 years (ref.)	1.00	1.00	1.00	1.00
	70-79 years	1.46***	1.42***	1.20***	1.22***
	80 years or +	2.02***	2.06***	1.26***	1.30***

Sex	Male (ref.)	1.00	1.00	1.00	1.00
	Female	1.18***	1.09**	1.01	0.99

Place of residence	Rural (ref)	1.00	1.00	1.00	1.00
	Urban	1.02	1.09**	1.09**	1.04

Level of education	Higher than secondary (ref.)	1.00	1.00	1.00	1.00
	Secondary level	1.66***	1.59***	1.48***	1.47***
	Primary/elementary level	2.33***	2.35***	2.12***	2.01***
	No schooling	2.47***	2.54***	2.13***	2.02***

Marital status	Married (ref.)	1.00	1.00	1.00	1.00
	Separated/divorced	1.22*	1.22*	1.16	1.09
	Widowed	1.03	1.00	0.95	0.92**
	Single	1.23**	1.28***	1.24**	1.24**

Working status during the past 7 days	Work (ref.)	1.00	1.00	1.00	1.00
	Do not work	1.80***	1.61***	1.36***	1.36***

Average total income per year	100,000 Baht or more (ref.)	1.00	1.00	1.00	1.00
	30,000-99,999 Baht	1.17***	1.22***	1.23***	1.17***
	10,000-29,999 Baht	1.38***	1.47***	1.45***	1.34***
	Less than 10,000 Baht	1.78***	1.93***	1.80***	1.62***

Living arrangement	Living with children (ref.)	1.00	1.00	1.00	1.00
	Spouse	.97	0.94	0.97	0.95
	Alone	.86**	0.88*	0.90*	0.88*
	Other	.94	0.94	0.95	0.94

Number of chronic diseases	No disease (ref.)	-	1.00	1.00	1.00
	1 disease	-	2.00***	1.89***	1.83***
	2 diseases	-	2.93***	2.57***	2.43***
	3 or more diseases	-	5.51***	4.03***	3.71***

Functional Status	Able to do all ADLs (ref.)	-	-	1.00	1.00
	Difficulty in 1 and 2 ADLs	-	-	1.79***	1.71***
	Difficulty in 3 or more ADLs	-	-	3.71***	3.32***

Psychosocial symptoms	No symptom (ref.)	-	-	-	1.00
	1-2 symptoms	-	-	-	1.57***
	3-4 symptoms	-	-	-	2.19***
	5 or more symptoms	-	-	-	2.68***

**Intercept**		.23***	.17***	.19***	0.14***

**-2 Log likelihood**		39265.5	37940.6	36815.4	35995.5

**Cox & Snell R Square**		0.077	0.116	0.148	0.171

Note: *** = p < .001, ** = p < .01, * = p < .05

## Discussion

The principal determinants of self-assessed poor health of the elderly population in Thailand found in this study were chronic diseases, functional limitations, and psychosocial symptoms. The number of chronic diseases is one of the clearest determinants of self-assessed poor health in almost all studies conducted among the elderly [6,20,21,31]. This was confirmed in our study population with an adjusted odds ratio of 3.70 for an increase of 3 and more chronic conditions compared to those who had no chronic disease. The functional limitation is another principal determinant of self-assessed poor health. In particular, the adjusted odds ratio is 3.3 among those who had difficulty in 3 or more ADLs. The chronic disease and functional limitation were also major determinants of self-assessed health of the elderly population of Spain [21]. Psychosocial symptoms are the third important determinant of self-assessed poor health among Thai elderly, with an adjusted odds ratio of 2.7 among those who had 5 and more symptoms. However, a study conducted in Netherlands, did not find association between SAH and psychosocial factors [18]. In case of Thai elderly when the chronic diseases were included in the model 2 it was affecting the reporting of poor health around 6 times more likely. But when functional status and psychosocial symptoms were included in model 3 and model 4 the OR of chronic diseases reduced to 3.7 times.

In this study, age, education, marital status, working status, socioeconomic condition and living arrangement also have significant effects on self-assessed poor health. Age is a significant indicator of self assessment of poor health in this study. The oldest group perceived their health to be poorer than did the younger group. The finding matches other studies conducted among the older people of Thailand, as well as among indigenous Australians and older people in Singapore and Iran [11,19,22,32]. This can be explained by the fact that ageing causes difficulty in physical movement and in the ability to carry out the basic ADLs independently [11]. However, it was found that the self perception of health was better among the oldest group compared to the youngest group and seems to be a reflection of the normal adaptation process or acceptance of comorbidity and disabilities as normal in the ageing process [21]. Subjective health worsens gradually, but only slightly, between ages 65 and 85 years. This could be due to the fact that these individuals were hardy survivors [15]. Jylha (2009) mentions this association of self-assessed health and age as a universal phenomenon [2]. We found that the people who had education higher than secondary they were less likely to report poor health. Educated people are more aware of diseases, their consequences, and the utilization of health services in comparison to less educated or illiterate people [21]. We also found that those who were separated/divorced (OR: 1.09) and single (OR: 1.24) were more likely to report poor health compared to married people. This finding matches with the study conducted by Zimmer and Amornsirisomboon in Thailand [25] and another study done by Tajvar Arab and Montazeri in Iran [32]. The elderly people who live single they have low social participation, higher level of loneliness due to a lack of emotional support within the household as well as an absence of practical support [33-35]. However, under the living arrangement we have found that those who live alone are less likely to report poor health. They might have good health and they can help themselves in daily activities. Socioeconomic status is a strong predictor of self-assessed health in this study, just as in other studies [5,7,32,36,37], and can influence health outcomes by affecting access to medical care, the ability to fulfill one's basic needs, participation in the society, enjoyment of life, freeing one from worry about life's emergencies and unexpected future expenses for the elderly [35]. The influence of sex on perception of health is somewhat mixed. Although in our study sex was not a significant predictor, other studies found that it was an important predictor. Studies in Spain and Thailand found that females were less likely to report their health as good than were males [11,21,24]. However, sex did not significantly influence perception of health among the older people of Singapore [22]. But SAH became a stronger predictor of mortality for men compared to women [18,33,34].

The proportion of variance of self-assessed health explained in this study was quite low. There might be specific determinants of self-assessed health in the Thai population that were not covered by the sociodemographic and health-related variables included in this study. This study has identified multiple predictors of self-assessed health of older people of Thailand. Two previous studies [24,25] conducted in Thailand did not include psychosocial symptoms as predictors of self-assessed health of older people. The current study found that the psychosocial variable has a significant effect on people's perceptions concerning their own health at old age. The study used nationally representative samples. The results can be generalized to the self-assessment health of Thai older people. However, the study has certain limitations. Since it was a cross-sectional study, we could not see cause-effect relationships. Though psychosocial symptoms were examined in order to measure the real effect of this variable detailed questions need to be tested. The functional status could also be examined in more detail, including basic activities of daily living, activities of daily living and instrumental activities of daily living. Moreover, to have deeper understanding the psychosocial symptoms and activities under functional status should be validated among the Thai elderly in local context. Further research is needed to investigate the SAH of Thai elderly.

## Conclusion

Self-assessed poor health is not uncommon among the older people in Thailand. No single factor accounted for the self-assessed poor health; multiple factors contributed in this regard. Among them, the study found that chronic diseases, functional status, and psychosocial symptoms were the strongest determinants of self-assessed poor health among elderly people living in Thailand. From this study it can be concluded that programs should aim to improve the health status of older people of Thailand by focusing on all these identified issues so that the overall well-being of the ageing population can be improved.

## Competing interests

The authors declare that they have no competing interests.

## Authors' contributions

FH, RA, KS conducted data analysis, interpreted the data, and drafted the manuscript. All authors read and approved the final manuscript.

## Pre-publication history

The pre-publication history for this paper can be accessed here:

http://www.biomedcentral.com/1471-2318/10/30/prepub
